# Matrix bound nanovesicles from decellularized pericardium facilitate angiogenesis

**DOI:** 10.3389/fbioe.2026.1980409

**Published:** 2026-10-16

**Authors:** Dalila Di Francesco, Carolina Di Varsavia, Irene Regano, Luca Di Nunno, Simona Casarella, Nausicaa Clemente, Diego Mantovani, Francesca Boccafoschi

**Affiliations:** 1 Laboratory for Biomaterials and Bioengineering, Canada Research Chair Tier I for the Innovation in Surgery, Department of Min-Met-Materials Engineering and Regenerative Medicine, CHU de Quebec Research Center, Laval University, Quebec, QC, Canada; 2 Department of Health Sciences, University of Piemonte Orientale, Novara, Italy; 3 Settore Centri di Ricerca e Infrastrutture di Ateneo e Laboratori - Polo di Novara, University of Piemonte Orientale, Novara, Italy

**Keywords:** angiogenesis, decellularized extracellular matrix, endothelial cells, matrix bound nanovesicles, pericardium, regenerative medicine

## Abstract

Therapeutic angiogenesis is a regenerative medicine approach which aims to induce revascularization for tissue repair. Among the existing strategies used to promote angiogenesis, biomaterials derived from decellularized tissues and composed of extracellular matrix (dECM) possess intrinsic angiogenic and regenerative potential. Recently, a bioactive component of such ECM materials has been identified; specifically, matrix bound nanovesicles (MBV). MBV have been shown to possess highly bioactive and tissue-specific cargo, and the ability to induce a potent immunomodulatory and regenerative response. However, their angiogenic potential has not been studied yet. Herein, the angiogenic properties of MBV derived from bovine pericardia dECM is evaluated. MBV show cytocompatibility toward endothelial cells, enhancing their functional phenotype and inducing the expression of angiogenic markers. In the presence of MBV, endothelial cells form three-dimensional monolayered endothelial vessel-like structures in tube formation assay. MBV also enhance endothelial sprouting in aortic ring assay. Finally, in hind limb ischemia *in vivo* model, MBV prevent tissue necrosis and restore vascularization and tissue function. These results demonstrate the angiogenic potential of MBV from pericardia ECM and represent a promising approach for therapeutic angiogenesis in regenerative medicine applications.

## 1 Introduction

Angiogenesis, the formation of new blood vessels from pre-existing vessels, is an essential process for functional tissue repair and successful wound healing. However, many pathologies characterized by ischemia, such as chronic wounds and myocardial infarction, can lead to delayed or impaired tissue repair and loss of tissue function (Shi et al., 2023). For these reasons, inducing revascularization in ischemic conditions represents a global clinical interest, and in the context of regenerative medicine, angiogenesis represents a critical consideration for all therapeutic strategies (Henry, 1999; Bian et al., 2019; Veith et al., 2019; Li et al., 2020; Guan et al., 2021; Wang et al., 2021; Zhang et al., 2021; 2022; Wariyar et al., 2022; Yan et al., 2022; Zheng et al., 2022). However, functional revascularization of ischemic tissue is not an easy task, given that physiological angiogenesis is a particularly complex and dynamic process. In fact, angiogensis occurs through a multistep process which includes: an initial endothelial cell response to angiogenic factors, focal degradation of the basement membrane for the sprouting of new vessels, migration and proliferation of endothelial cells, tubulogenesis, vessel maturation and stabilization, and finally the synthesis of new basement membrane. These steps are normally coordinated by finely-tuned mechanisms which include cell-cell interaction, ECM-cell interaction, enzymes, specifc signaling pathways and angiogenic factors (DiPietro, 2016; van Hinsbergh, 2017; Ionescu et al., 2022).

Various strategies have been developed and explored for therapeutic angiogenesis, including the delivery of angiogenic factors, biomaterial mediated angiogenesis, stem cell therapy and gene therapy (Chu and Wang, 2012; Augustine et al., 2019; Veith et al., 2019; Giraudo et al., 2020; Ngo and Harley, 2020). Still, these approaches present limitations, such as achieving effective targeted angiogenesis, and other safety concerns, thus their clinical translation is hampered, and the clinical need for angiogenic strategies remains (Augustine et al., 2019; Johnson et al., 2019).

Interestingly, among materials used in regenerative medicine, those composed of naturally occurring ECM have been shown to possess innate angiogenic potential (Golebiowska et al., 2024). ECM-based hydrogels and scaffolds have shown great potential for therapeutic angiogenesis, demonstrating ability to intrinsically carry and deliver angiogenic factors, sustain endothelial cell functions, and promote angiogenesis *in vivo* (Rajabi-Zeleti et al., 2014; Alvarèz Fallas et al., 2018; Jeong et al., 2018; Rajabi et al., 2018; Seo et al., 2018; Bracey et al., 2019; Fu et al., 2019; Di Francesco et al., 2022; Cui et al., 2023; Wang et al., 2023). In fact, these materials demonstrated excellent bioactivity in different tissue regeneration contexts. The mechanism of action of ECM-based materials is still not fully understood, however their biological potential has been attributed to the retention of physiological components able to modulate cellular processes for tissue regeneration, such as cytokines, chemokines and structural proteins (Hoganson et al., 2010; Parmaksiz et al., 2016; Hussey et al., 2018; Heath, 2019; Brown et al., 2022). Additionally, a recent discovery has brought insight on the bioactive effect of ECM materials: the presence of matrix bound nanovesicles (MBV).

MBV are a subtype of extracellular vesicle (EV) embedded within the collagen component of ECM, rather than being found in body fluids like other EV, and have been successfully isolated from different decellularized ECM (dECM) tissues (Huleihel et al., 2016; Turner et al., 2022; Piening and Wachs, 2023; Francesco et al., 2025). In spite of their relatively recent discovery, the bioactivity of these nanovesicles is clearly evident, and their potential for regenerative medicine applications is robust (Faust et al., 2017; Van Der Merwe et al., 2019; Crum et al., 2022; 2023a; 2023b; Turner et al., 2022; Campbell et al., 2024; Kobayashi et al., 2024; Liao et al., 2024; Cramer et al., 2025). Indeed MBV demonstrated therapeutic potential in applications such as rheumatoid arthritis, acute inflammatory conditions, central nervous system repair, and osteolytic environments (Faust et al., 2017; Van Der Merwe et al., 2019; Crum et al., 2022; 2023a; Liao et al., 2024). The biological advantage of MBV appears to be related to their rich cargo and cell signaling capability, their ability to induce a powerful immunomodulatory effect and to recapitulate the safety and efficacy of their source dECM material (Huleihel et al., 2017; Hussey et al., 2019; 2020; Cramer et al., 2022; Turner et al., 2022; Capella-Monsonís et al., 2025). MBV show to reproduce the immunomodulatory effect of parent dECM materials, suggesting a role in dECM biomaterials‘ bioactivity (Huleihel et al., 2017; Turner et al., 2022). However, whether they also contribute to other biological effects of dECM, such as angiogenic potential, remains unknown. In fact, the angiogenic potential of MBV has not yet been evaluated (Huleihel et al., 2016; Piening and Wachs, 2023).

The aim of the present research is to determine the angiogenic potential of MBV. To this aim, MBV are derived from decellularized bovine pericardium (dBP), a biomaterial used in regenerative medicine which has demonstrated intrinsic angiogenic ability (Seif-Naraghi et al., 2010; 2011; Umashankar et al., 2013; Jara et al., 2015; Mazzitelli et al., 2015; Olsen et al., 2016; Alhadrami et al., 2019; Shklover et al., 2019; Zouhair et al., 2020; Carton et al., 2021; Xing et al., 2021; Di Francesco et al., 2022; 2024b; Eyuboglu et al., 2024). The presence of MBV in dBP was also recently reported (Di Francesco et al., 2024a). Therefore, to determine if dBP-derived MBV retain angiogenic potential, their ability to induce endothelial cell functionality, expression of angiogenic markers, tube formation, endothelial sprouting and to treat *in vivo* limb ischemia, were investigated.

## 2 Materials and methods

### 2.1 Matrix-bound nanovesicles isolation

Decellularized bovine pericardium extracellular matrix (dBP) was kindly provided by Tissuegraft Srl (Alessandria, Italy) (Italian patent number 102025000018355, PCT number PCT/IB2026/057331, submitted July 2026). Briefly, 10 mg/mL of lyophilized and ground dBP was enzymatically digested with pepsin [1 mg/mL] (Sigma-Aldrich, P7012, Milan, Italy), for 65 h at room temperature in acidic buffer HCl 0.01 M. Solubilized dBP was neutralized to physiological pH (7.4) and salinity using NaOH and PBS, then underwent differential centrifugations, starting with a 100 x g centrifugation for 5 min, then a 10000 x g centrifugation for 90 min at 4 °C. Then, the supernatant was ultracentrifuged at 100000 x g for 70 min at 4 °C (Optima™ LE-80K ultracentrifuge, Beckman Coulter, Brea, CA, United States). Finally, the MBV pellet was resuspended in particle-free phosphate buffered saline (PBS) or cell specific culture medium and filtered with 0,22 μm pore filters (Sartorius, 17823-K, United States). Resuspended samples were stored at −20 °C until further use. Nanovesicle concentration and size distribution were determined by nanoparticle tracking analysis (NTA) and transmission electron microscopy (TEM), as previously described (Di Francesco et al., 2024a) (Supplementary Figure S1). MBV were isolated at concentration of 12*10^14^ MBV per 100 mg of dBP. For all experiments, MBV were used at a concentration of 6*10^14^ MBV/mL. For all biological replicates, MBV were isolated from pericardia obtained from different bovine donors.

### 2.2 Cell culture

Human umbilical vein endothelial cells (HUVEC) were purchased (ATCC, CRL-1730, VA, United States) and maintained in Kaighn’s Modification of Ham’s F-12 Medium (F-12K) (ATCC 30-2004, VA, United States), supplemented with 0.1 mg/mL heparin (Sigma Aldrich, Milan, Italy), 0.06 mg/mL endothelial cell growth supplement (ECGS) (Corning, Berlin, Germany), 1% penicillin, streptomycin, and amphotericin-B (PSF) (Euroclone, Nordhausen, Germany) and 10% fetal bovine serum (FBS) (Gibco, Milan, Italy). Cells were used up to the 6^th^ passage.

### 2.3 Cell viability

The cytocompatibility of MBV towards HUVEC was evaluated using MTS assay (Promega Italia Srl, Milan, Italy). HUVEC were seeded in 96-well plates at a concentration of 15000 cells/well. MBV resuspended in cell medium were added 24 h after cell seeding, to ensure cellular adhesion to the well. Control condition was represented by cell cultured in maintenance medium without MBV. At time points of 1 and 3 days, MTS assay was performed by discarding culture media and adding 250 μL MTS solution, composed of 4:5 DMEM without phenol red (Cytiva SH30585.02, Fisher scientific, Milan, Italy) and 1:5 MTS solution, according to manufacturer’s protocol. The 490 nm absorbance was read using Victor 4X Multilabel Plate Reader (Perkin Elmer, Milan, Italy) and data were analyzed in Excel (Microsoft, Redmond, WA, United States). Experiments were conducted as biological triplicates, each time with technical triplicates.

### 2.4 Cellular uptake of MBV

MBV membranes were labelled using the ExoGlow™-Membrane EV Labeling Kit (EXOGM600A-1, System Biosciences, California, United States), according to manufacturer’s protocol. Labelled MBV were then filtered with 0.22 μm pore filters and resuspended in PBS. For cellular uptake, HUVEC were seeded in 48-well plates at a concentration of 10000 cells/well in F-12K 10% FBS medium. 24 h after cell seeding, 100 μL of labelled MBV were added in the medium of each well. At 24h, the medium was removed from the well. Both cells and cell medium containing labelled MBV were visualized using an EVOS™ FLoid™ Cell Imaging Station (Thermo Fisher Scientific, Milan, Italy). Experiments were conducted as biological triplicates.

### 2.5 vWF immunofluorescence staining

MBV effect on HUVEC morphology and phenotype was evaluated using vWF-DAPI immunofluorescence staining. Cells were seeded in 24-well plates over microscope glass slides, at a concentration of 100000 cells/well. After 24h, MBV resuspended in cell medium were added to the wells. Control condition was represented by cells cultured in maintenance medium without MBV. At time points of 1 and 3 days, samples were fixed using 4% formaldehyde for 1 h at room temperature. Fixed samples were permeabilized with a 0.5% Triton X-100 solution for 5 min and blocked with a 3% BSA solution for 20 min. Samples were then incubated for 1 h with anti-vWF antibody (Rabbit, polyclonal, Ab6994, Abcam, Milan, Italy), and a secondary anti-rabbit antibody Alexa Fluor™ 546 (goat, A-11035, Invitrogen, Massachusetts, United States) for 1 h. Nuclei were stained with 1 μg/mL DAPI (Sigma-Aldrich, Milan, Italy) for 1 min. Stained samples were imaged using anLSM800 confocal microscope (Carl Zeiss, Germany). To evaluate relative vWF fluorescence, representative images for experimental triplicates were analyzed using ImageJ software. Fluorescence value was normalized over the cell number counted in each image. Experiments were conducted as biological triplicates, each time with technical triplicates.

### 2.6 Human angiogenesis antibody array

Human Angiogenesis Antibody Array (Abcam ab134000, Milan, Italy) is an antibody pair-based assay. Capture antibodies are supplied arrayed/spotted on a membrane, with each pair of spots representing a different analyte, and are as follows: Angiogenin, Epidermal Growth Factor (EGF), C-X-C motif chemokine 5 (ENA-78), basic Fibroblast Growth Factor (bFGF), Growth-Regulated Oncogene (GRO), Interferon-gamma (IFN-γ), Insulin-like Growth Factor-1 (IGF-I), IL-6, IL-8, Leptin, Monocyte Chemoattractant Protein-1 (MCP-1), Platelet Derived Growth Factor (PDGF-BB), Placental Growth Factor (PlGF), Chemokine (C-C motif) ligand 5 (RANTES), Transforming Growth Factor-beta (TGF-beta1), Tissue inhibitors of metalloproteinases 1 and 2 (TIMP-1 and TIMP-2), Thrombopoietin, VEGF-A and VEGF-D. HUVEC were seeded at a concentration of 100000 cells/well in 24-well plates and cultured for 1 and 3 days with MBV. Control condition was represented by cells cultured in maintenance medium without MBV. At 1 and 3 days, samples were lysed according to manufacturer’s protocol and protein concentration was quantified using bicinchoninic acid (BCA) protein assay kit (Novagen®, BCA Protein Assay Kit, 71285-3, Madison, WI, United States). 50 μg of cell lysates were used for human angiogenesis antibody array according to the producer’s protocol. The membranes were then analysed at ChemiDoc (Bio-Rad, Hercules, CA, United States) for chemiluminescence detection. The comparison has been performed using ImageJ for a semi-quantitative analysis, and signals were normalized towards positive control spots on the CTR day 1 membrane array. Experiments were conducted as biological duplicates, each time with technical duplicates.

### 2.7 Western blot

Western blot was performed to evaluate eNOS expression in HUVEC cultured with MBV. For cell lysate samples, 100000 HUVEC/well were seeded in 24-well plates. 24 h after cell seeding, MBV resuspended in cell medium were added to the wells. Control condition was represented by cells cultured in maintenance medium without MBV. At time points of 1 and 3 days, cell lysis for protein extraction was performed by combination of chemical, RIPA buffer, and mechanical, cell scraping, methods. Protein quantification of cell lysates was then performed with Pierce BCA Protein Assay Kit (Thermo Fisher, Milan, Italy) according to the producer’s protocol. 10 μg of cell lysate samples were loaded in a 10% N,N′-Methylenebisacrylamide (acrylamide) (MerckMillipore 01709, Darmstadt, Germany) running gel for SDS-PAGE run. For molecular weight standard, the prestained protein Sharpmass VI marker (Euroclone, Milan, Italy) was also loaded in the gel. After the electrophoretic run, Western blot transfer was performed. The nitrocellulose membranes were incubated overnight with rabbit anti-eNOS, 130 kDa (MerckMillipore, SAB4502014, Darmstadt, Germany) and mouse anti-tubulin, 55 kDa (MerckMillipore, 05-829, Darmstadt, Germany) primary antibodies, followed by 1 h incubation with the secondary anti-rabbit and anti-mouse antibodies conjugated with Horse Radish Peroxidase (HPR) (Perkin Elmer, Milan, Italy). Chemiluminescence detection was performed using Western Lightning Plus Enhanced Chemiluminescence Substrate kit (ECL) (Perkin Elmer, Milan, Italy). The membranes were then analyzed at ChemiDoc (Bio-Rad, Hercules, CA, United States) for chemiluminescence detection. For densitometry analysis the Image Lab (Version 6.0.1; Bio-Rad, Hercules, CA, United States) software was used. Values were normalized using tubulin expression. Experiments were conducted as biological triplicates, each time with technical triplicates.

### 2.8 Zymography

Zymography was performed to evaluate MMPs expression in HUVEC cultured with MBV. For cell lysate samples, 100000 HUVEC/well were seeded in 24-well plates. 24 h after cell seeding, MBV resuspended in cell medium were added to the wells. Control condition was represented by cells cultured in maintenance medium without MBV. At time points of 1 and 3 days, cell lysis for protein extraction was performed by combination of chemical, RIPA buffer, and mechanical, cell scraping, methods. Protein quantification of cell lysates was performed with Pierce BCA Protein Assay Kit (Thermo Fisher, Milan, Italy) according to the producer’s protocol. 10 μg of cell lysate samples were loaded in a 7.5% N,N’-methylenebisacrylamide (acrylamide) (MerckMillipore 01709, Darmstadt, Germany) running gel, containing 0.2% gelatin (Sigma-Aldrich, G8150, Milan, Italy) for SDS-PAGE run. For molecular weight standard, the pre-stained protein Sharpmass VI marker (Euroclone, Milan, Italy) was also loaded in the gel. After the electrophoretic run, gels were washed in 2.5% Triton X-100 (Sigma-Aldrich, Milan, Italy) for 3 h and incubated overnight in an ionic buffer, (50 mм Tris-HCL pH: 7.4, 10 mм CaCl_2_, 150 mм NaCl). Gels were fixed for 2 h in a 40% methanol, 10% acetic acid buffer, and stained with blue Coomassie 0.05% (Sigma-Aldrich, Milan, Italy) for 1 h. Gel destaining was performed in until necessary and gel images were acquired using ChemiDoc (Bio-Rad, Hercules, CA, United States) for Coomassie gel detection. For densitometry analysis the Image Lab (Version 6.0.1; Bio-Rad, Hercules, CA, United States) software was used. Experiments were conducted as biological triplicates, each time with technical triplicates.

### 2.9 Tube formation assay

Tube formation assay was performed using collagen type I as a substrate material. 50 μL of collagen type I solution were prepared in 48-well plates and allowed to gel for 30 min at 37 °C. HUVEC were seeded on the gels at a concentration of 40000 cells/well, and F-12K 10% FBS medium was added. MBV resuspended in cell medium were added right after cell seeding. Control condition was represented by cells cultured in maintenance medium without MBV, on collagen type I coatings. At time points of 2 h and 24 h, images of tube formation were acquired using an inverted phase contrast microscope. At 24 h the medium was removed, and samples were fixed for 1 h using 4% formaldehyde at room temperature. Samples were stained for immunofluorescence with either anti-CD31 antibody-DAPI or with phalloidin-DAPI. For anti-CD31 fluorescent staining, fixed samples were permeabilized with a 0.5% Triton X-100 solution for 5 min and blocked with a 3% BSA solution for 20 min. Samples were then incubated for 1 h with anti-CD31 antibody (mouse, monoclonal, MA513188, Invitrogen, Massachusetts, United States), and a secondary anti-mouse antibody Texas Red-X (goat, T-6390, Invitrogen, Massachusetts, United States) for 1 h. Nuclei were stained with 1 μg/mL DAPI for 1 min. For phalloidin staining, Phalloidin-Fluorescein Isothiocyanate Labeled (Sigma-Aldrich, P5282, Milan, Italy) was incubated on the samples for 45 min at 37 °C. Nuclei were stained with 1 μg/mL DAPI for 1 min. Phalloidin stained samples were observed using a fluorescence microscope (Leica DM700, Wetzlar, Germany), and images were acquired via Leica software LAS V4.7 (Leica, Wetzlar, Germany). CD31 stained samples were observed and acquired using an EVOS™ FLoid™ Cell Imaging Station (Thermo Fisher Scientific, Milan, Italy). Experiments were conducted as biological triplicates, each time with technical triplicates.

### 2.10 Aortic ring assay

Organ explants have been authorized by Ministero della Salute (Notification n◦ DB064.N.TMC). The mice were bred under pathogen-free conditions in the animal facility of the University of Piemonte Orientale, Department of Health Sciences (Authorization n◦ 217/2020-PR) and treated in accordance with the Ethical Committee and European guidelines. To perform aortic ring assay, aortas were isolated from BALB/c male mice. Aortic ring assay was performed according to Kapoor et al.’s method (Kapoor et al., 2020). Briefly, the explanted aortas were washed 5 times in serum free F-12K 10% medium. Aortas were cleaned from fat and 1 mm rings were cut from aorta with the aid of a stereomicroscope. Each ring was placed in a 48-well plate well on a previously gelled 150 µL Collagen Type I coating. After ring placement, additional 50 µL of collagen were added on top of the ring, and allowed to gel for 30 min at 37 °c. After, F-12K 2.5% FBS medium containing MBV was added in the wells, and F-12K 2.5% FBS medium was used as control condition. Cell media was refreshed every 5 days. At time points of 0, 7 and 14 days images were acquired using an inverted phase contrast microscope to evaluate cell sprouting from aortic rings. Images were then analyzed using ImageJ software to evaluate sprouting area, each value was normalized towards the respective aorta’s area.

Aortic rings at day 14 were fixed using 4% formaldehyde for 1 h at room temperature. Fixed samples were permeabilized with a 0.5% Triton X-100 solution for 5 min and blocked with a 3% BSA solution for 20 min. Samples were then incubated for 1 h with anti-human CD31 antibody (mouse, monoclonal, MA513188, Invitrogen, Massachussets, United States), and then with secondary anti-mouse antibody Texas Red-X(goat, T-6390, Invitrogen, Massachussets, United States) for 1 h. Nuclei were stained with 1 μg/mL DAPI (Sigma-Aldrich, Milan, Italy) for 1 min. Stained samples were imaged using an EVOS™ FLoid™ Cell Imaging Station (Thermo Fisher Scientific, Milan, Italy). Experiments were conducted as biological triplicates.

### 2.11 Hind limb ischemia *in vivo* model

The *in vivo* experiments have been authorized by Ministero della Salute (Authorization n◦ 779/2021-PR Risp. a prot. DB064.73). The mice were bred under pathogen-free conditions in the animal facility of the University of Piemonte Orientale, Department of Health Sciences (Authorization n◦ 217/2020-PR) and treated in accordance with the Ethical Committee and European guidelines. To assess the angiogenic and regenerative potential of MBV, the HLI *in vivo* model was used (Brenes et al., 2012; Ungerleider et al., 2016). 12 BALB/c male mice were anesthetized by an intraperitoneal injection of Zoletil® + Xylazine (47.5 mg/kg and 16 mg/kg, respectively). Animals were placed on a heated pad and a humectant eye gel was applied for the entire time of the surgery. The femoral artery was isolated from other structures with the help of a stereomicroscope and was cauterized with a thermal cautery unit. On the cauterized femoral artery, 50 μL of MBV were added (3 × 10^13^ MBV), and an intramuscular injection of the same quantity was also performed in the gracilis muscle of the cauterized limb for 6 mice. 50 μL of PBS were instead injected in 6 mice for control conditions. Afterwards, the skin was stitched with adsorbable suture 5.0 and the mice were kept under a red hot lamp until full awakening. Mice were placed into singular cages and checked daily up to 28 days to assess their ability to use the limb and the possible presence of necrosis. A functional score was given based on visual evaluation according to the Tarlov scale and modified ischemia scale (Brenes et al., 2012). Evaluation and scoring was performed in a blind manner. On day 28, mice were sacrificed to obtain a limb specimen for histological evaluation. Histological sample preparation was performed after 4% formalin fixation for a day. Samples were dehydrated, cleared and embedded in paraffin, and 5 μm thick sections were obtained. The sections were then deparaffinized, rehydrated, and stained with hematoxylin-eosin (H&E stain) for light microscopy observation. The experiment was performed in sextuplicate for all the tested conditions. To evaluate relative blood vessel presence in injection area 28 days after treatment, representative images of all samples were analyzed using ImageJ software.

### 2.12 Statistical analysis

Sample means and standard deviations were calculated for all datasets. Statistical significance was assessed using a one-tailed Student’s t-test assuming equal variances, performed in InStat3 (GraphPad Instat Software Inc., United States), with a significance threshold of α = 0.05. A one-tailed test was selected because the hypothesis was directional *a priori*, effects in the opposite direction were not considered biologically meaningful. Pairwise comparisons were conducted either between control and MBV-treated samples or between different time points within the same experimental condition. Results with p < 0.05 were considered statistically significant.

## 3 Results

### 3.1 MBV biocompatibility towards endothelial cells

The cytocompatibility of MBV towards HUVEC was evaluated using cell viability assay. Results (Figure 1A) demonstrate that MBV have a lower cell viability at day 1 compared to the control (CTR), however, by day 3 MBV induce a significant increase in cell viability, compared both to day 1 and to CTR at day 3. Moreover, Figure 1B shows that by 24 h MBV are internalized by HUVEC, as highlighted by black arrows, without inducing cytotoxic effects.

**FIGURE 1:**
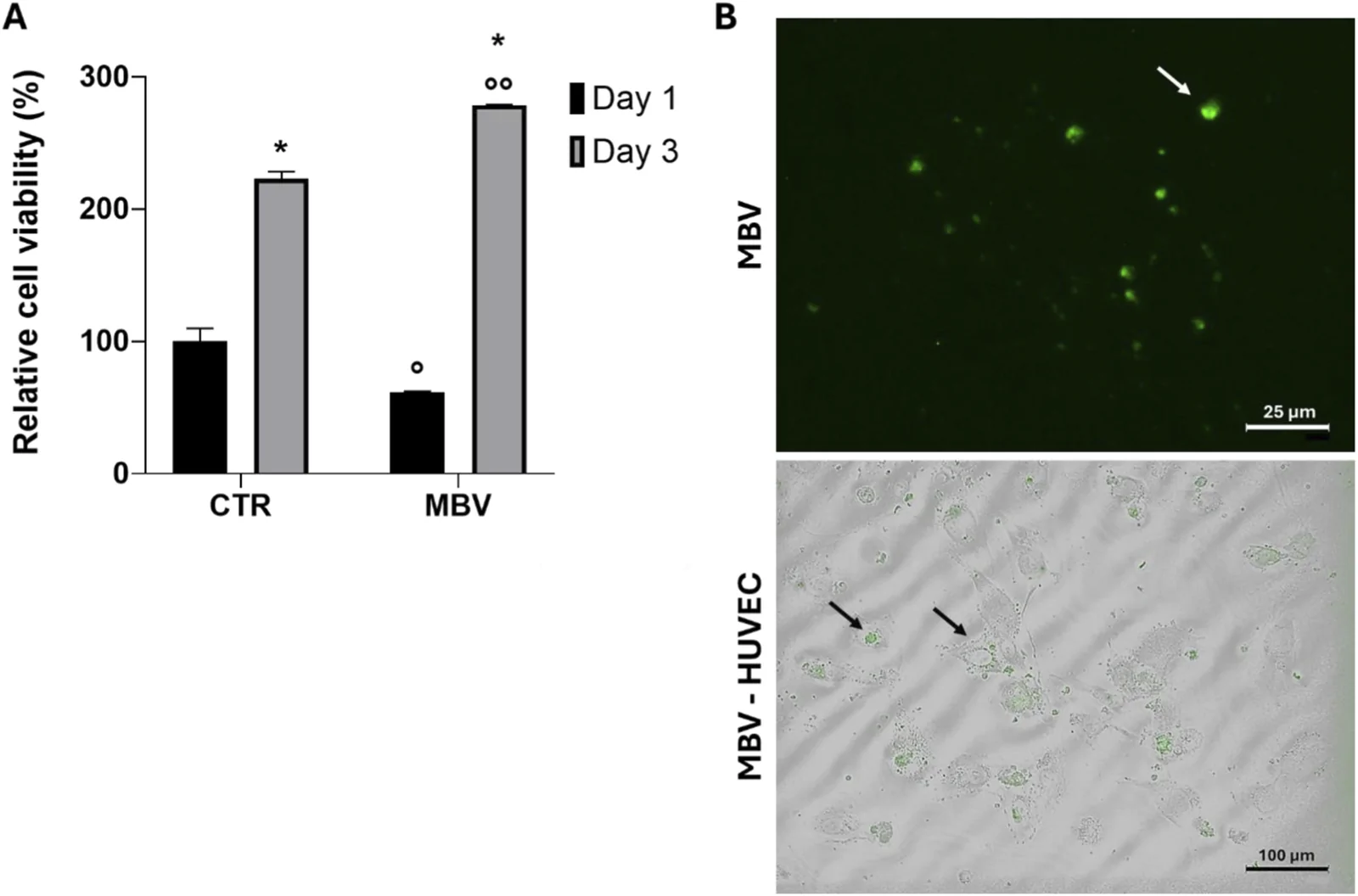
MBV cytocompatibility and internalization in endothelial cells. **(A)** Cell viability of HUVEC cultured with MBV for 1 and 3 days. *p < 0.001 days 3 vs. day 1; ° p < 0.05 MBV vs. CTR; ° p < 0.01 MBV vs. CTR. **(B)** Fluorescent MBV (green) in cell medium and internalization of fluorescent MBV (green) in HUVEC after 24 h of cell culture. Arrows indicate fluorescent MBV.

The cytocompatibility of MBV is also confirmed with endothelial phenotype, as there are no morphological alterations when comparing HUVEC in CTR or MBV condition at day 1 (Figure 2A). At this time point, both conditions express similar amounts of endothelial marker von Willenbrand Factor (vWF) (Figure 2B). By day 3, HUVEC cultured in presence of MBV showed a noticeable network formation. This correlates to a statistically significant increase in vWF expression with respect to CTR and to day 1.

**FIGURE 2:**
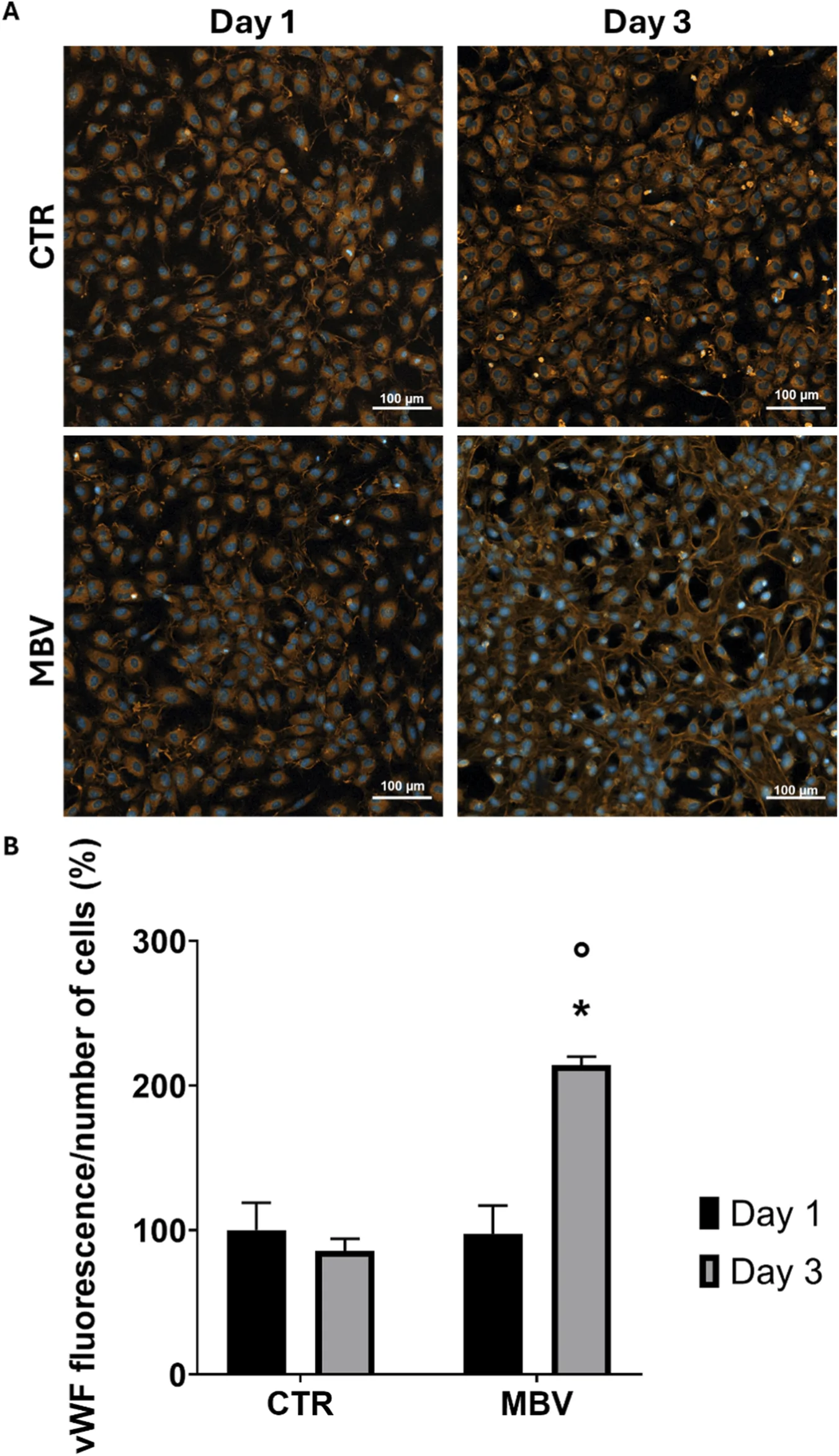
vWF expression in endothelial cells cultured with MBV for 1 and 3 days. **(A)** Representative immunofluorescence staining imaging of vWF (red) and nucleus-DAPI (blue) in HUVEC. **(B)** Relative quantification of vWF fluorescence over cell number, normalized towards CTR at day 1. *p < 0.001 days 3 vs. day 1; ° p < 0.001 MBV vs. CTR.

### 3.2 MBV enhance the expression of angiogenic markers in endothelial cells

The expression of endothelial angiogenic marker nitric oxide synthase (eNOS) was evaluated by Western blot method (Figure 3). Results show that, on day 1 of cell culture, MBV maintain a comparable expression of eNOS to CTR. However, MBV induce a statistically significant increase in eNOS expression at 3 days compared to both day 1 and to the CTR condition.

**FIGURE 3:**
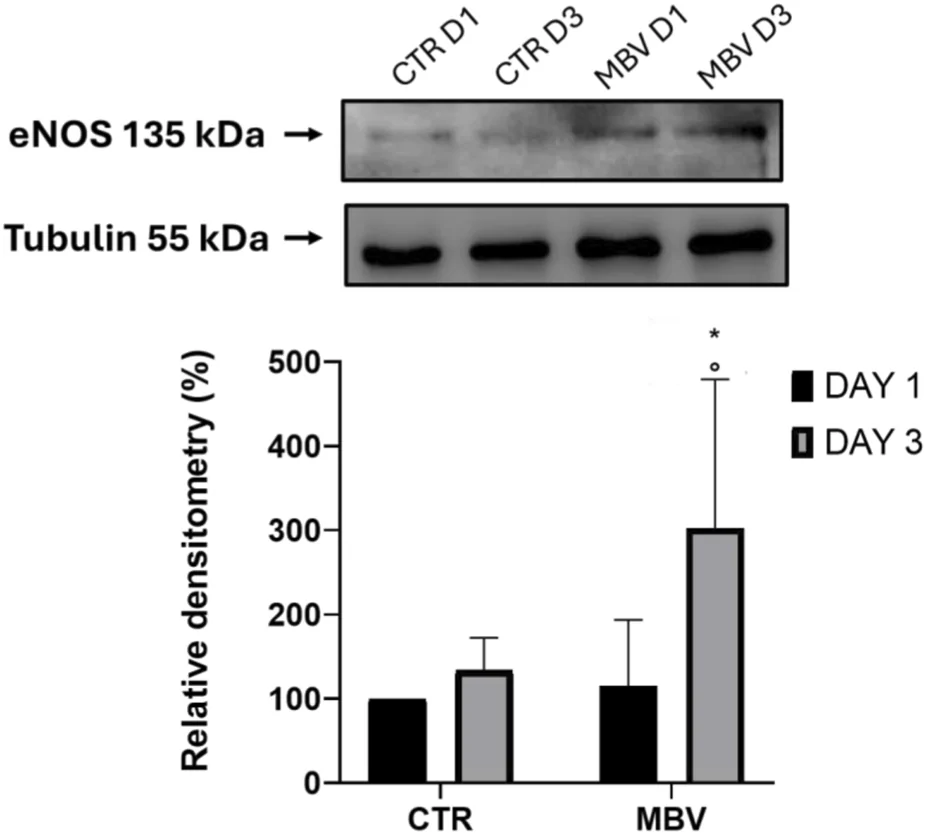
eNOS expression in endothelial cells cultured with MBV for 1 and 3 days. Western blot analysis of eNOS and tubulin (house-keeping protein) expression in HUVEC cultured with MBV. Relative densitometry is presented as normalized towards CTR at day 1. *p < 0.05 days 3 vs. day 1; ° p < 0.05 MBV vs. CTR.

The expression of angiogenic metalloproteinases (MMPs) was also evaluated by gelatin zymography (Figure 4). In this case also, MBV induce a time-dependent increase in MMP2, as a statistically significant higher expression of active MMP2 is obtained from day 1 to day 3, strengthening the CTR trend.

**FIGURE 4:**
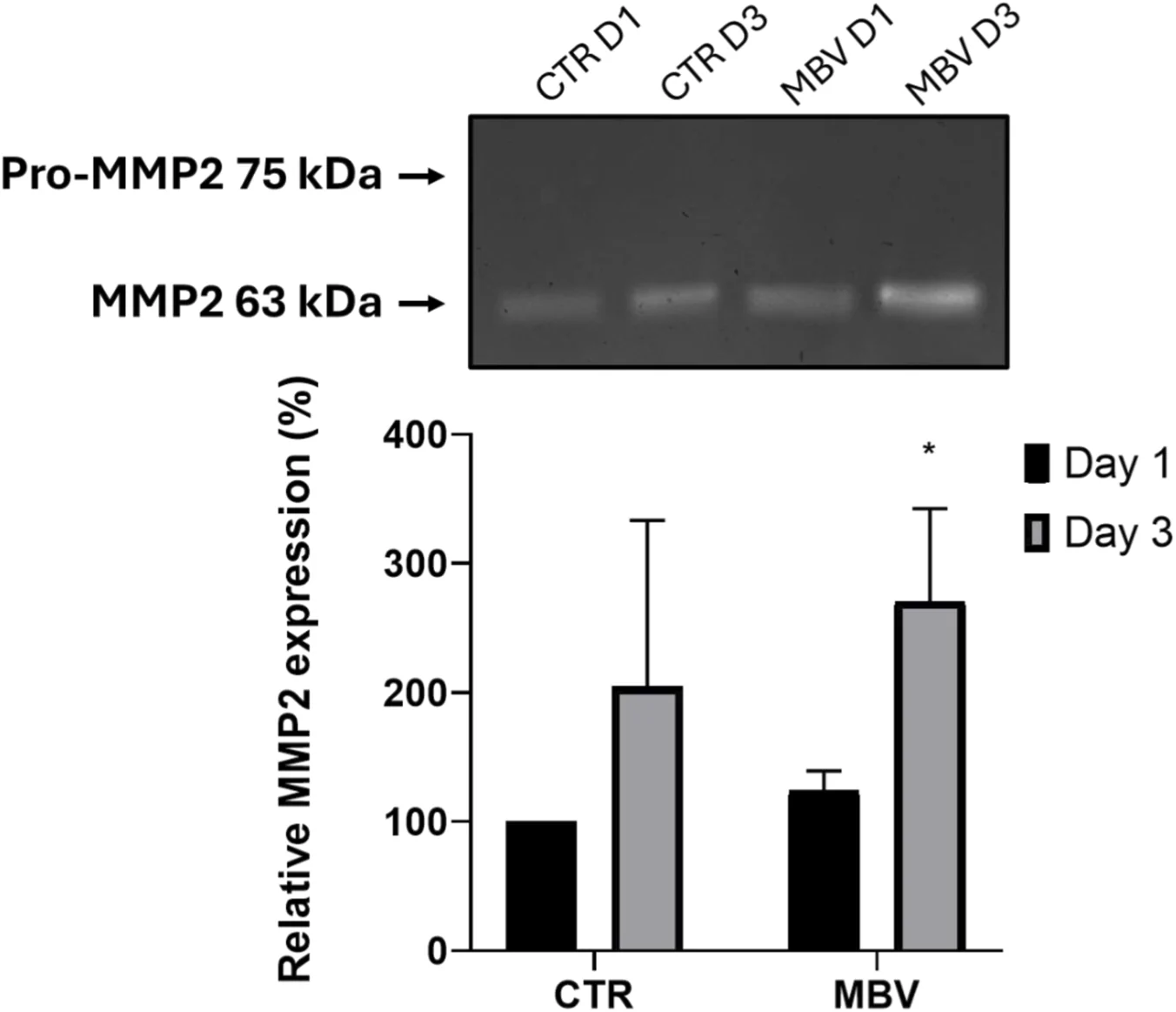
Metalloproteinases expression in endothelial cells cultured with MBV for 1 and 3 days. Zymography analysis of MMP2 forms expression in HUVEC cultured with MBV. Relative expression is presented as normalized towards CTR at day 1. *p < 0.05 days 3 vs. day 1.

### 3.3 MBV induce the formation of 3D tubular endothelial structures

To evaluate MBV’s angiogenic potential, tube formation assay was performed. Both the CTR and MBV conditions did not show any tubule-like structures (i.e., branches and nodes) (Figure 5A). However, in 2 h, MBV stimulation induced the formation of thick, multicellular structures in the wells, measuring up to a few millimeters in length. By 24 h, these formations showed three-dimensionality, which was also confirmed by immunofluorescence staining (Figure 5B), where mono-layer endothelial tubular structures can be observed. Looking at the immunofluorescence staining for endothelial adhesion molecule CD31 (also known as PECAM-1) in the tubular structures induced by MBV, HUVEC clearly express CD31 on the surface of interacting cells, while little to no CD31 is expressed in the CTR.

**FIGURE 5:**
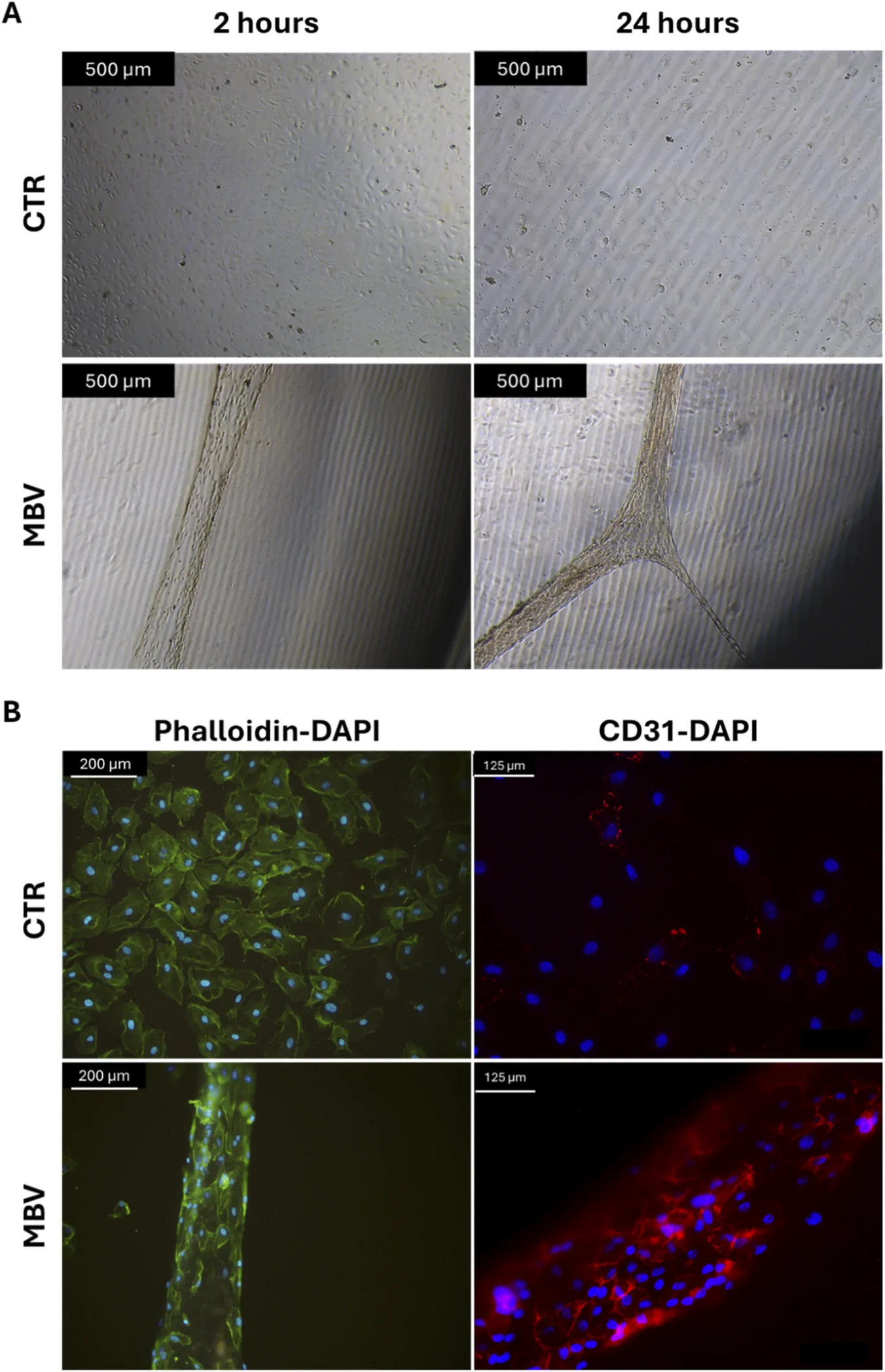
Tube formation assay of endothelial cells cultured with MBV. **(A)** Representative phase contrast images of tube formation assay at 2 and 24 h. **(B)** Representative immunofluorescence staining imaging of cytoskeleton actin-Phalloidin (green), CD31 (red) and nucleus-DAPI (blue).

### 3.4 MBV enhance endothelial cell sprouting in aortic ring assay

Aortic ring assay was performed to analyze endothelial cell sprouting induced by MBV stimulation. Representative images show a clear and increased sprouting in aortas cultured in the presence of MBV (Figure 6A). A cellular network of sprouted endothelial cells can be observed in the MBV condition. The quantification of sprouting area reveals no differences between CTR and MBV at day 7, while by day 14, MBV induced a significantly larger sprouting area compared to CTR (Figure 6B).

**FIGURE 6:**
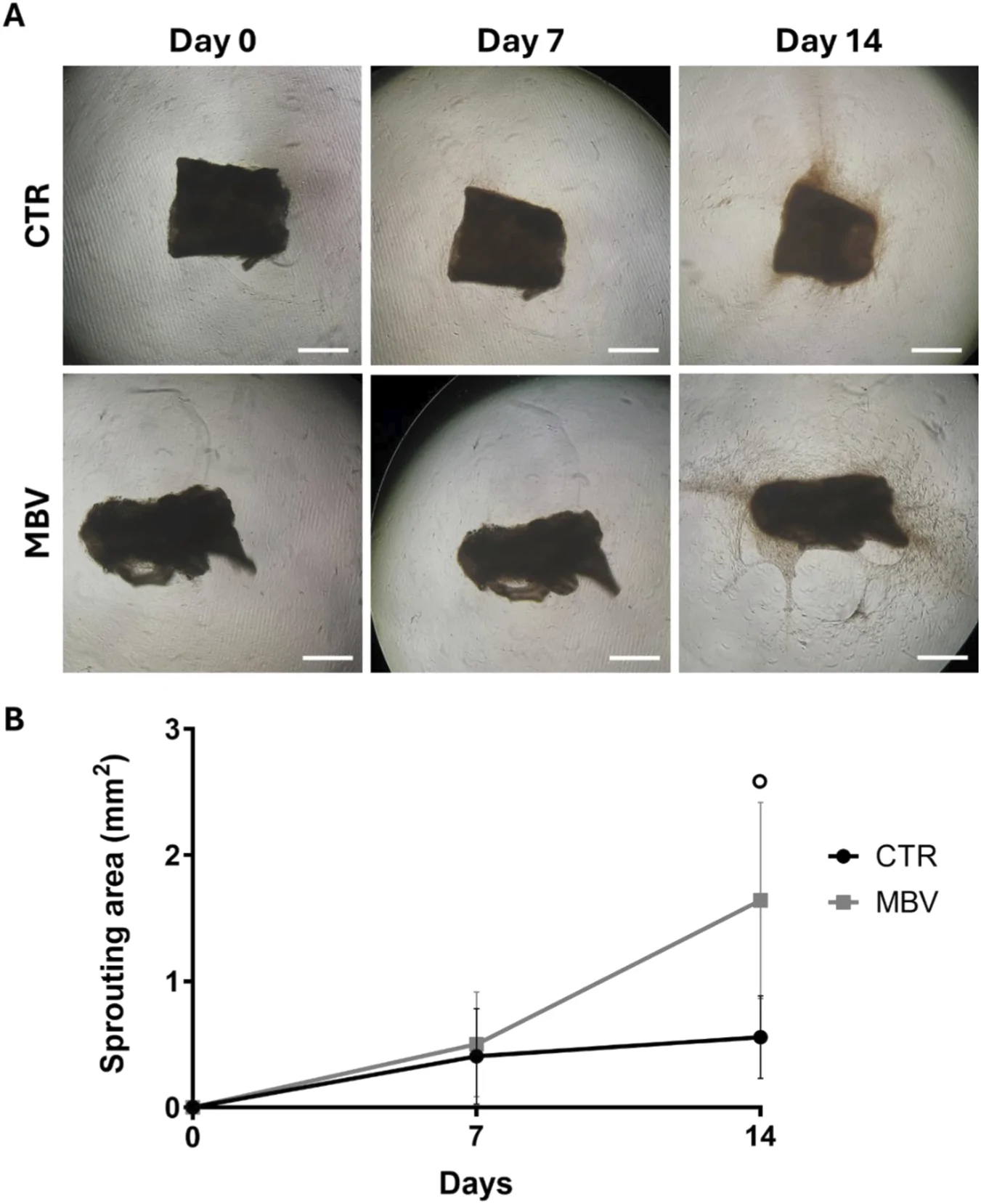
Endothelial sprouting under MBV stimulation in aortic ring assay. **(A)** Representative phase contrast images of aortic rings. Scale bar 500 µm. **(B)** Quantification of sprouting area of aortic rings. ° p < 0.01 MBV vs. CTR.

### 3.5 MBV induce functional regeneration in hind limb ischemia model

The hind limb ischemia (HLI) model was used to evaluate the *in vivo* angiogenic and regenerative potential of MBV. Limbs’ functionality and morphological features were quantified using the Tarlov and Modified ischemia score respectively (Brenes et al., 2012). Results indicate that the damaged limb was able to regain significant functionality, in terms of ability to walk, within 14 days from MBV treatment (Figure 7A). 1 month after MBV injection, the models fully regained limb functionality, while CTR groups remained unable to walk normally. Morphologically, limbs treated with MBV did not display limb alterations and did not develop tissue necrosis at any level (Figure 7B), while CTR groups developed necrosis leading to the loss of different fingers (Figure 7C). No alterations were evidenced in MBV treated group even at day 1 after ischemic damage. Histologically, MBV treated limbs at 1 month show no differences to healthy tissue, and no inflammation in the injection area, suggesting a full tissue recovery; instead, CTR groups presented tissue alterations and a remarkable inflammatory infiltrate (Figure 7D). Histological sections of the MBV treated condition show the formation of new healthy blood vessels, which are comparable in quantity to those found in native limb tissue, the CTR condition instead shows a significantly lower number of blood vessels (Figure 7E).

**FIGURE 7:**
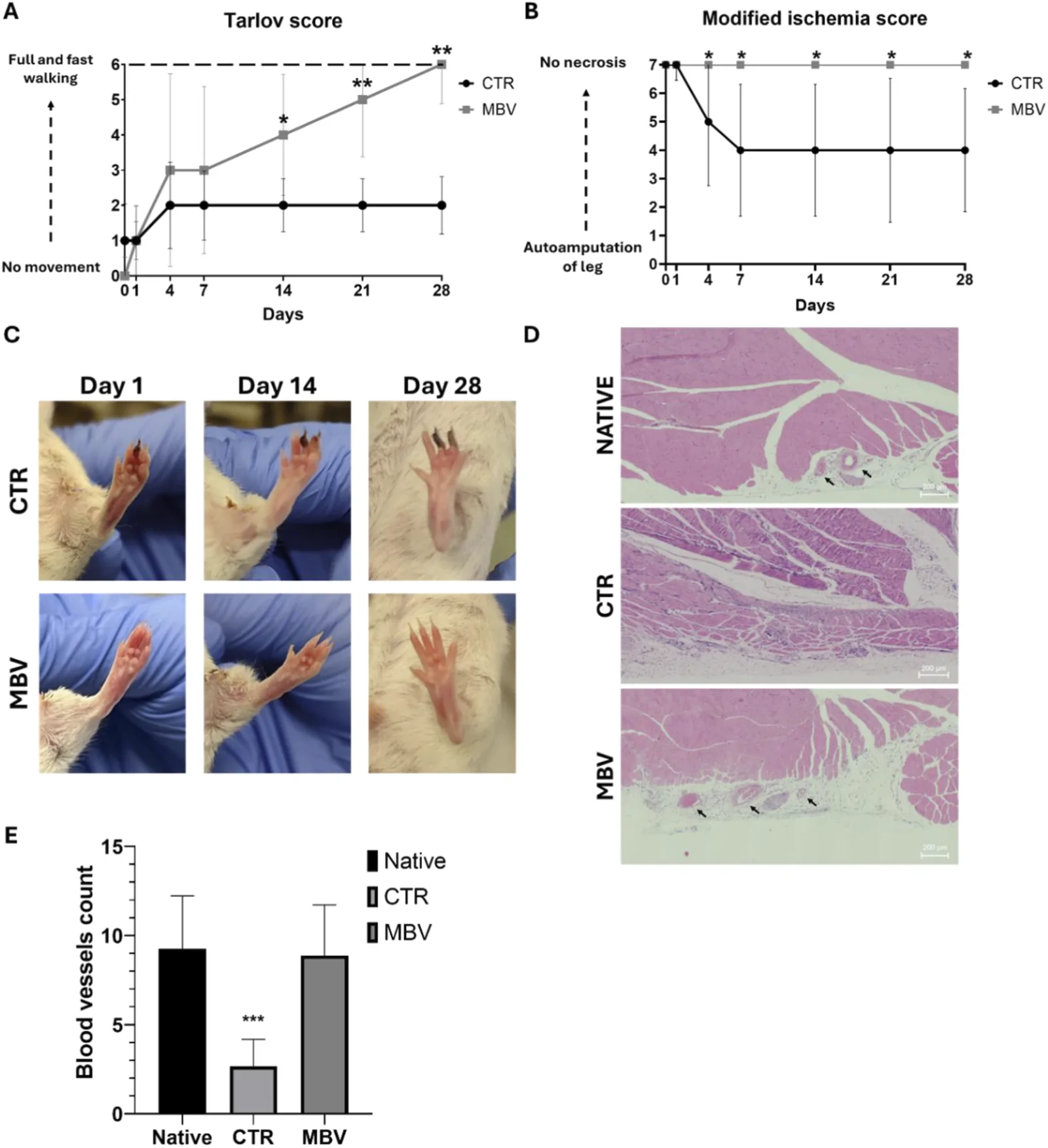
MBV effect on HLI *in vivo* model. **(A)** Tarlov score of HLI model, scoring functionality of treated limb from day 0 to day 28. *p < 0.05 MBV vs. CTR; **p < 0.001 MBV vs. CTR. **(B)** Modified ischemia score, scoring morphological appearance of treated limb from day 0 to day 28. *p < 0.05 MBV vs. CTR. **(C)** Representative images of morphological appearance of HLI models. **(D)** Histological images of tissue sections of models’ limb injection area (injection site from the bottom-right side of the pictures). Arrows highlight the presence of blood vessels. **(E)** Quantification of blood vessels present in histological sections of native limbs and CTR and MBV treated HLI limbs. ***p < 0.005.

## 4 Discussion

The results of the present study show that MBV derived from dBP facilate angiogenesis by inducing the production of angiogenic factors, the formation of 3D endothelial vessel-like structures and endothelial cell sprouting, and the promotion of therapeutic revascularization and functional tissue repair in ischemic conditions.

Endothelial cells cultured with different MBV concentrations show a time-dependent increase in cell viability, significantly higher than that of the CTR (Supplementary Figure S2). Even though a decreased endothelial metabolic activity is recorded during the first 24 h after MBV administration, the other results demonstrate the biocompatibility of MBV. In fact, an increase in cell viability is then evidenced over time, even after MBV are internalized by the cells, confirming the lack of MBV cytotoxicity. These results correlate to those reported in literature, as other studies show a quick internalization of MBV in different cell types without any alteration of cell functions (Huleihel et al., 2017; Crum et al., 2023b). Kobayashi et al., reported the compatibility of MBV isolated from another tissue towards endothelial cells, demonstrating MBV ability to induce endothelial cell proliferation (Kobayashi et al., 2022). When examining the morphology of endothelial cells, no phenotypical differences are observed with respect to the control 24 h after MBV stimulation, confirming a healthy endothelial phenotype in presence of MBV. By 3 days of cell culture, MBV induce the formation of an endothelial cellular network, reflecting in a significantly higher expression of vWF, not reported in the control condition. The ability of endothelial cells to interact with each other and form networks is one of the first steps of the angiogenesis pathway (Li et al., 2011; Hsu et al., 2021), usually occurring in presence of a substrate/support (Arnaoutova et al., 2009; Nagamori et al., 2013; DeCicco-Skinner et al., 2014). In the present study, we show the ability of MBV to spontaneously generate an early cellular network between endothelial cells, despite the absence of a matrix-like support.

The ability of MBV to stimulate the initial steps of angiogenesis is also confirmed through the enhancement of angiogenic markers eNOS and MMP2. Results indicate that MBV prompt a time-dependent and significant increase in the production of eNOS with respect to control, which is important both for endothelial homeostasis and angiogenesis (Namba et al., 2003; Heiss et al., 2015). The ability of different factors and/or materials to induce the activation of the eNOS pathway for angiogenesis has proven beneficial in innumerable defective conditions, like ischemic ones, providing the correct environment for tissue repair (Bir et al., 2012; Yu et al., 2020; 2024; Liao et al., 2021; Nguyen et al., 2021; Zhou et al., 2022). In presence of MBV, the expression of the active form of MMP2 was also increased from day 1 to day 3, strengthening the trend observed in the control. MMPs are important factors in tissue remodeling and essential for angiogenesis and tissue repair (Rojiani et al., 2010; Wolosowicz et al., 2024). The upregulation of MMP2 in rapid timings has been shown to be correlated to angiogenic effects (Chuang et al., 2015; Sun et al., 2017; Liu et al., 2018; Ren et al., 2022). Therefore, these results imply that MBV can activate early endothelial pathways involved in angiogenesis. Supplementary results (Supplementary Figure S3) indicate that this activation is complex and involves many factors, as other angiogenic markers are expressed differentially by endothelial cells cultured with MBV. For example, the expression of monocyte chemoattractant protein-1 (MCP-1), tissue inhibitors of metalloproteinases (TIMPs) and fibroblast growth factor (FGF), is increased by MBV, although not significantly. Of these, MCP-1 and FGF represent potent angiogenic factors (Yamada et al., 2003; Niu et al., 2008; Farooq et al., 2021; Ornitz and Itoh, 2022), while TIMPs have anti-angiogenic roles (Moore and Crocker, 2012). The production of TIMPs is however essential in conditions where MMPs are upregulated. This suggests that MBV do not simply induce uncontrolled angiogenesis, but that a fine-tuned and complex endothelial response ensues under MBV stimulation (Löffek et al., 2011; Moore and Crocker, 2012; Kremastiotis et al., 2021; Zhou et al., 2021).

Tube formation assay was performed using collagen type I as substrate, instead of other traditional basement membrane extracts, to avoid providing angiogenic stimuli other than those presented by MBV. Tube formation was no achieved in the control condition, correlating to what is reported in literature, as tube formation on collagen gels is generally altered or slowed (Kroon et al., 2002; Arnaoutova et al., 2009, 20). Tube-like structures were instead formed as early as 2 h after MBV stimulation on collagen gels. During *in vitro* tube formation, the endothelial network formed is commonly immature, with thin tubule structures that present only intracellular lumens (Nakatsu et al., 2003; DeCicco-Skinner et al., 2014). Instead, we herein demonstrated that under MBV stimulation, millimeter-long and thick 3D endothelial structures are formed within 24 h. The monolayer vessel-like structure of endothelial cells formed by MBV induction is highlighted with actin immuno-staining. These structures resemble the intima layer of capillaries, in which endothelial cells closely interact with each other, as shown by the evident expression of CD31 on their membrane. CD31 expression in MBV-induced endothelial structures further validates the angiogenic roles of MBV (Cao et al., 2002; Privratsky and Newman, 2014; Park et al., 2015).

The aortic ring assay is a more physiologically relevant platform to evaluate the angiogenic potential of a stimulus, as it recapitulates different angiogenic steps, such as matrix degradation, cell migration, proliferation, sprouting and branching, and formed vessels stability (Baker et al., 2012; Kapoor et al., 2020). Aortic rings cultured with MBV showed, not only enhanced sprouting from aortas, but a significantly wider area of vessel outgrowths, which included branching in the sprouting area. Thus, MBV enhanced all aforementioned steps of angiogenesis, inducing clear matrix degradation, cell migration and the formation of a complex sprouting network, which included endothelial networks expressing CD31 (Supplementary Figure S4).

Finally, the potential of MBV was validated in an *in vivo* ischemic model, since HLI models are commonly used to assess biomaterials‘ angiogenic potential in terms of novel blood vessel formation and evaluation of functional tissue repair (Brenes et al., 2012; Aref et al., 2019; Kim et al., 2023). In this context, injections of MBV in the ischemic area demonstrated to prevent the formation of tissue necrosis with a significant vascularization of the interested tissue. Tissue necrosis and inflammatory infiltrate led to the loss of limb’s functionality in the control condition. Instead, MBV treatment allowed to significantly recover limb functionality 2 weeks after treatment, with a full function recovery 1 month after treatment.

Research on MBV is still early, and there is much to be discovered, such as MBV mechanism of action and bioactive roles. Other EV types, such as exosomes and microvesicles, have been well characterized instead in the past years, and their role in angiogenesis has been reported (Todorova et al., 2017; Ateeq et al., 2024). In general, EV have demonstrated to modulate angiogenesis in both physiological and pathophysiological conditions, due to their ability to intervene in angiogenic pathways, and induce angiogenesis, especially in tumor development (Kumar et al., 2024). Nevertheless, literature reports the study of EV for therapeutic angiogenesis in regenerative medicine, demonstrating their beneficial effects, such as in myocardial infarction models or osteogenic repair applications (Bian et al., 2014; 2019; Kang et al., 2015; Zhang and Huang, 2021; Cheng et al., 2023; Ma et al., 2023). Although the angiogenic effect of EV has been demonstrated, there are still doubts on the exact mechanisms with which EV induce angiogenesis. One explanation could be the presence of different angiogenic factors, such as proteins and miRNAs, which are present in EV and produced under specific conditions (Muralidharan-Chari et al., 2010; Amini et al., 2018; Bian et al., 2019). In this context, the tissue-specificity of EV seems to greatly contribute to their cargo and biological performance, and could also play a role in the angiogenic potential (Li et al., 2021; Turner et al., 2022). A tissue-specific role in the angiogenic potential of MBV reported herein could also be hypothesized. Different materials derived from pericardium have shown angiogenic ability such as the one reported in the present study, suggesting that angiogenic cues could also come from the tissue source. Beltrami *et al.*, reported of pericardial fluid derived exosomes containing specific miRNAs able to induce angiogenesis and functional vascular repair (Beltrami et al., 2017). Hassanbad *et al.* demonstrated the angiogenic properties of EV present in the pericardial fluid (Fatehi Hassanabad et al., 2026). Rajabi-zeleti *et al.*, also found that their pericardial scaffolds induced angiogenesis *in vivo* (Rajabi-Zeleti et al., 2014). Dergilev *et al.*, describe that mesothelial-like cells derived from pericardial space retain proangiogenic properties and have biological effects on endothelial cells (Dergilev et al., 2025). Seif-Naraghi *et al.*, demonstrated that injectable scaffolds derived from both human and porcine pericardium promoted neovascularization *in vivo* (Seif-Naraghi et al., 2010). Furthermore, we previously published on the angiogenic potential of a dECM hydrogel derived from the same raw tissue as the MBV described here (Di Francesco et al., 2022). These researches all point to a tissue-specific angiogenic property of pericardium, thought to be characteristic of the pericardial space and the inner mesothelial lining of the serous pericardium. The studies also suggest an angiogenic cell signalling perpetrated by the EV released by the mesothelial cells of the pericardium, in both the pericardial fluid, and in the pericardial extracellular matrix, as suggested by the results reported herein. Hence, this research validates both the angiogenic role of pericardial material and the fact that MBV alone are able to recapitulate it.

Albeit the fact that the ability of MBV to facilitate angiogenesis is herein demonstrated, the mechanism of action remains unknown and should be further investigated, representing a limitation of this study. Mechanisms of action reported for other EV types describe that their biological effects are orchestrated in a complex way which includes different pathways (Liu and Wang, 2023). Internalization of the vesicles and cargo release, either by endocytosis, micropynocytosis or membrane fusion, has been confirmed as a central mechanism of EV cell signalling. Cargo content is what then drives the subsequent biological response. Our previous research, characterizing the protein and cytokine content of dBP-derived MBV, showed the presence of different factors that may contribute to angiogenesis, such as FGFs (Di Francesco et al., 2024a). Thus, The MBV cargo may partially explain MBV bioactivity and angiogenic potential. However, other literature reports that both EV and MBV effects are also partly reliant on the presence of specific microRNAs, which were not evaluated in this study (Huleihel et al., 2016; 2017; Beltrami et al., 2017; Dellett et al., 2017; Barghchi et al., 2021; Piening and Wachs, 2023). Finally, EV have also shown to induce their biological effects through receptor-ligand cell signalling, which was not explored herein, but is theorized to be a mechanism included in MBV mode of action (Cerrotti et al., 2024).

Another limitation is the lack of additional purification of MBV, which may have led to co-isolation of other bioactive factors found in decellularized extracellular matrix. This needs to be further addressed in the future, and standardized guidelines for MBV isolation, like those described for the isolation of other EV, will need to be defined.

Angiogenesis has shown to be an essential part of tissue regeneration, and its potential was confirmed in many applications, such as myocardial regeneration, wound healing, and bone regeneration (Augustine et al., 2019; Yegappan et al., 2019; Cheng et al., 2020; Jahani et al., 2020; Munarin et al., 2020; Song et al., 2024; Chávez et al., 2025). Furthermore, materials with angiogenic properties have shown numerous benefits in the regenerative medicine context (Jung and Kleinheinz, 2013; Augustine et al., 2019; Jahani et al., 2020; Ngo and Harley, 2020). The present research highlights both the ability of MBV to facilitate angiogenesis, but also the therapeutic potential of MBV for tissue regeneration. Different research studies have demonstrated the bioactive role of MBV, and given their rich cargo, nanosize and proven pre-clinical safety, MBV rose as a novel attractive option regenerative medicine applications (Huleihel et al., 2016; Turner et al., 2022; Crum et al., 2023b; Piening and Wachs, 2023; Ayus-Martinez et al., 2024; Cramer et al., 2025). These premises have translated in a number of successful MBV applications in differnt biomedical contexts (Faust et al., 2017; Van Der Merwe et al., 2019; Crum et al., 2022; 2023a; Liao et al., 2024). Although the safety and potential of MBV have been demonstrated (Crum et al., 2023b; Capella-Monsonís et al., 2025), there is still work to do before considering their clinical potential, such as better understanding the mechanism of action, the possible scalability of these vesicles and their stability. In fact, when looking at other, more characterized EV types, such as exosomes which have been extensively researched for 40 years now, they are only now entering clinical trials (Rezaie et al., 2022), demonstrating that for the 10-year-old MBV there is still a long way to go.

In conclusion, the results of the present study show that MBV derived from dBP have an important angiogenic potential, which is a key process in tissue regeneration, homeostasis and development. Specifically, MBV maintain and enhance a functional endothelial phenotype, increase the expression of angiogenic markers, drive endothelial sprouting and prompt formation of 3D endothelial vessel-like structures. Finally, MBV prevent tissue necrosis and induce functional tissue regeneration in *in vivo* ischemic models. The therapeutic angiogenic boost provided by MBV makes them potential candidates for different regenerative medicine applications.

## Data Availability

The raw data supporting the conclusions of this article will be made available by the authors, without undue reservation.
