# Supplementary material for: Matrix bound nanovesicles from decellularized pericardium facilitate angiogenesis
Source: Front Bioeng Biotechnol. 2026 Oct 16;14:1980409. doi: 10.3389/fbioe.2026.1980409 (PMC13720949; doi:10.3389/fbioe.2026.1980409)
Supplement: Supplementary file 1 [file Table1.docx]

Supplementary Material

Matrix Bound Nanovesicles from Decellularized Pericardium Facilitate Angiogenesis

Dalila Di Francesco^1,2^, Carolina Di Varsavia^2^, Irene Regano^2^, Luca Di Nunno^1,2^, Simona Casarella^2^, Nausicaa Clemente^3^, Diego Mantovani^1^, Francesca Boccafoschi^2*^

^1^Laboratory for Biomaterials and Bioengineering, Canada Research Chair Tier I for the Innovation in Surgery, Department of Min-Met-Materials Engineering and Regenerative Medicine, CHU de Quebec Research Center, Laval University, Quebec, Canada

^2^Department of Health Sciences, University of Piemonte Orientale, Novara, Italy

^3^Settore Centri di Ricerca e Infrastrutture di Ateneo e Laboratori - Polo di Novara, University of Piemonte Orientale, Novara, Italy

*** Correspondence:**
Francesca BoccafoschiCorresponding Author
francesca.boccafoschil@med.uniupo.it

**Supplementary Figure 1.** Characterization of MBVs derived from dBP A) Representative concentration-size distribution of NTA analysis on MBVs is shown. B) Representative TEM images of MBVs fixed on carbon-coated grids. Scale bar 100 nm.

**Supplementary Figure 2.** Cytocompatibility of MBVs at different concentrations with endothelial cells. Cell viability of HUVECs cultured with MBVs for 1 and 3 days. MBV1 represents the highest concentration of 6*1014 MBVs/mL, while MBV2 represents the concentration 1.5*1014 MBVs/mL. * p < 0.001 day 3 vs day 1; ° p < 0.05 MBV vs CTR; °° p < 0.01 MBV vs CTR.

**Supplementary Figure 3.** Angiogenic cytokine expression of endothelial cells cultured with MBVs for 1 and 3 days. A) Human angiogenic cytokine antibody arrays imaging B) Relative densitometry values of main angiogenic cytokines expressed by HUVEC cells in antibody array. Values are presented as normalized towards respective CTR at day 1. C) Legend of spots presented in human angiogenic cytokine antibody array.

**Supplementary Figure 4.** CD31 expression in endothelial cell sprouting from aortic rings cultured with MBVs. Immunofluorescence staining imaging of CD31 (red) and nucleus-DAPI (blue) in endothelial cell sprouting from aortic rings at 14 days.
